# Next-generation liquid biopsies: detecting circulating epigenetic changes to identify translocation renal cell carcinoma

**DOI:** 10.1172/JCI201599

**Published:** 2026-02-02

**Authors:** Katsuhiro Ito, David A. Braun

**Affiliations:** Center of Molecular and Cellular Oncology, Yale Cancer Center, Yale School of Medicine, New Haven, Connecticut, USA.

## Abstract

Circulating tumor DNA detection in renal cell carcinoma has long been limited by the disease’s low DNA shedding. An aggressive subtype termed translocation renal cell carcinoma (tRCC) is notably more difficult to detect than the common type, clear-cell RCC, in part due to interindividual variability of gene fusions of the transcription factor TFE3, the driving factor in tRCC. In this issue of the *JCI*, Garinet et al. reported on an epigenomic liquid biopsy approach that identified a TFE3 fusion–associated chromatin signature specific to tRCC. This work demonstrated that fusion-driven epigenomic alterations can be captured noninvasively and used to distinguish tRCC from other renal cancer subtypes. Beyond its diagnostic potential, the approach described by Garinet et al. may enable disease monitoring and subtype classification in other genetically quiet tumors. Epigenomic liquid biopsy is a promising framework to improve diagnostic accuracy and guide personalized management for tRCC.

## Enhancing circulating tumor DNA detection in kidney cancer

Dead or dying tumor cells release fragments of DNA into the bloodstream, and these fragments are often termed circulating tumor DNA (ctDNA). Monitoring ctDNA has emerged as a noninvasive approach to various clinical applications such as early cancer diagnosis, assessment of minimal residual disease after surgery, and monitoring treatment response (1). While this approach has had notable success across many tumor types, including in a large prospective trial in bladder cancer (2), renal cell carcinoma (RCC) represents a particular challenge, as it exhibits the lowest ctDNA levels in plasma among extracranial tumors (3, 4). This low-shedding quality has limited the clinical application of ctDNA in RCC.

Initial efforts to improve detection focused on detecting somatic mutations in commonly altered genes in ctDNA from patients with RCC (5). However, due to the limited ctDNA release from these tumors, the sensitivity of conventional mutation-based assays was low. To address the limited sensitivity, patient-specific variant tracking approaches were developed (6). In this approach, the patient’s tumor is first sequenced to identify patient-specific somatic mutations and then a subset of those mutations can be tracked in the peripheral blood. More recently, epigenomic profiling of ctDNA has been explored to enhance detection sensitivity (7, 8). Epigenomic profiling captures cell-type–specific signatures such as changes in DNA methylation and chromatin modifications, which differ significantly between tumor and normal cells and can provide broader coverage of tumor-derived fragments. Through these advances, the sensitivity of ctDNA-based assays in RCC has improved substantially. However, most studies to date have predominantly focused on clear-cell RCC (ccRCC), which is by far the most common type of RCC. Data on non-ccRCC subtypes (sometimes called “variant” or “divergent” histologies) are substantially more limited.

Translocation RCC (tRCC) is a less common but aggressive subtype of RCC. tRCC is driven by MiT/TFE fusion oncoproteins, most commonly a *TFE3* fusion located on Xp11.2. Chromosomal translocation involving Xp11.2 generates *TFE3* gene fusions with more than 20 fusion partners, resulting in aberrant TFE3 activation and a distinct transcriptional pattern (9). Because of its morphological similarities to other RCC subtypes, tRCC has been often underdiagnosed (10, 11). Detection of ctDNA from tRCC using conventional approaches (detecting somatic single-nucleotide variants [SNVs], for example) is particularly challenging because these tumors typically have a lower tumor mutation burden (TMB) and MiT/TFE fusion breakpoints vary substantially among patients (12, 13). However, the detection of tRCC using a liquid biopsy approach may still be feasible. A recent study reported that an epigenomic liquid biopsy approach successfully detected sarcomatoid differentiation of RCC (14), suggesting that this approach might be able to capture non-SNV features of fusion-driven, low-TMB tumors such as tRCC. In this context, Garinet et al. (15) developed a tRCC-specific epigenomic signature from tRCC cell lines and demonstrated the accurate detection and monitoring of tRCC with plasma epigenomic (chromatin) profiling.

## Establishment of a TFE3 fusion–associated epigenomic signature

First, the authors defined a tRCC-specific epigenomic signature by profiling multiple TFE3-driven tRCC cell lines. They performed ChIP-seq for H3K4me3 and H3K27ac as well as methylated DNA immunoprecipitation and sequencing (MeDIP-seq) (16). H3K4me3 and H3K27ac are histone H3 modifications at lysine 4 and lysine 27, representing active promoters and active promoters/enhancers, respectively. In addition, MeDIP-seq identifies silenced promoter regions. The analysis revealed that tRCC exhibited unique histone modification patterns, while the CpG methylation pattern was comparable with that of ccRCC cell lines. Next, the investigators performed TFE3 ChIP-seq in both TFE3 WT non-RCC cell lines and tRCC cell lines to obtain TFE3 fusion–protein binding sites. They established a robust TFE3 fusion epigenomic signature by integrating these epigenomic data. When this signature was tested in plasma samples from patients with tRCC or ccRCC, and in healthy controls, it distinguished tRCC from ccRCC and from healthy controls. The authors also showed that the dynamics of tRCC-specific epigenomic signature score in plasma ctDNA were associated with treatment response and disease progression in tRCC patients.

**Figure 1:**
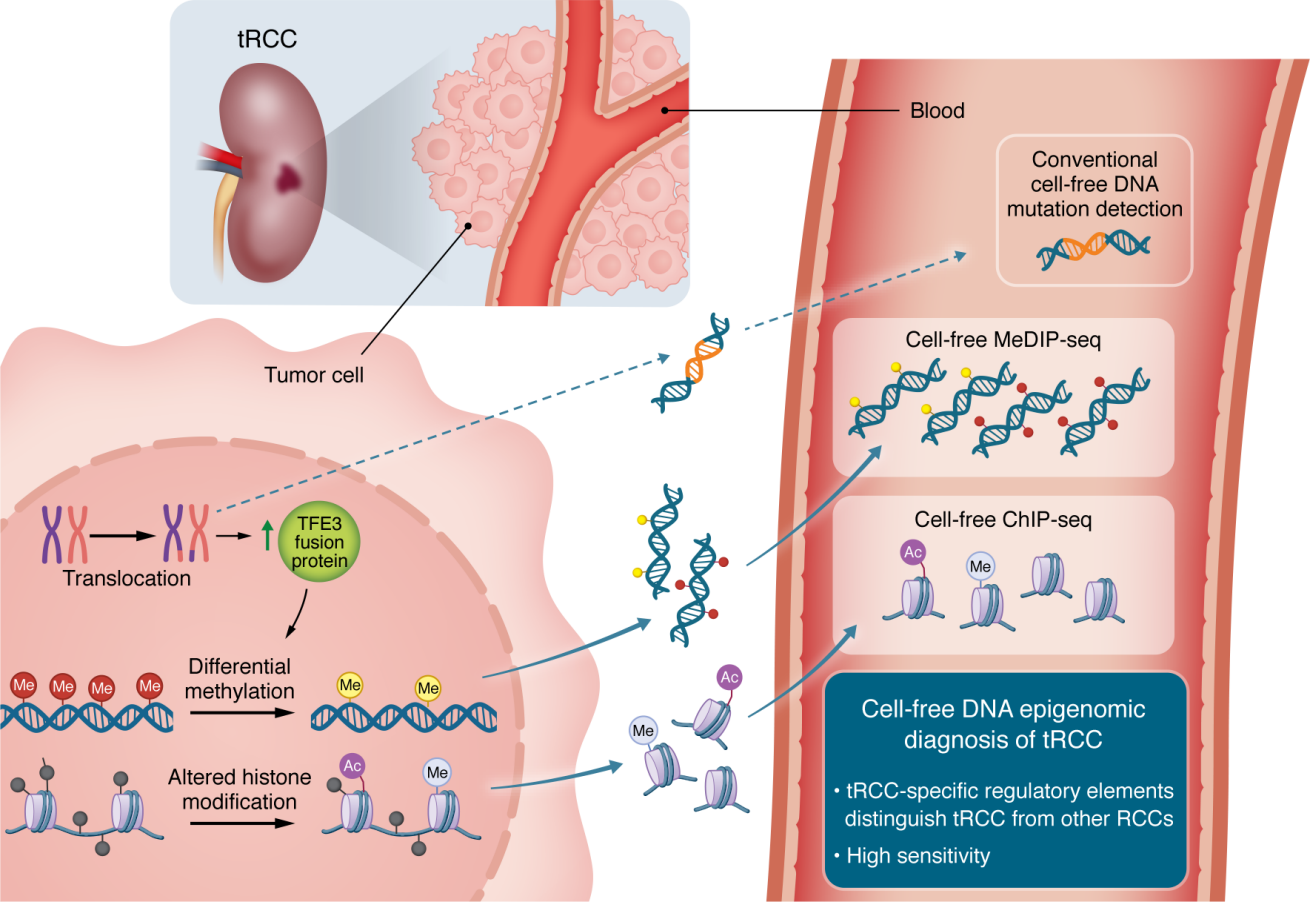
Cell-free DNA–based epigenomic diagnosis of tRCC. In tRCC, chromosomal translocation leads to the formation of a TFE3 fusion protein, which drives epigenomic alterations, including differential DNA methylation and histone modifications. Garinet et al.’s study demonstrated that these epigenetic changes can be detected in circulation as cell-free DNA or nucleosomes (15). Cell-free MeDIP-seq and histone ChIP-seq enabled the identification of tRCC-specific regulatory elements, allowing for sensitive detection and specific distinction of tRCC from other RCC subtypes.

This approach to detect a tRCC-specific epigenomic signature from plasma ctDNA is a promising tool for the diagnosis and disease management of tRCC (Figure 1). An epigenomic profiling approach is particularly useful if traditional imaging or tissue biopsy is challenging. The evaluation of epigenomic signature from ctDNA could be utilized for initial screening of tRCC, as tRCC can often masquerade histologically as other subtypes, leading to misclassification (10). Moreover, this approach could be expanded to other malignancies driven by few genetic events — for example, cancers driven by a single translocation, which typically have a low mutational burden and are difficult to detect using conventional ctDNA approaches. Overall, this study demonstrated that a single translocation event in tRCC induces dynamic epigenomic changes and that this epigenomic profile is identifiable in the peripheral blood using cell-free ChIP-seq assays.

## Future of epigenomic liquid biopsy in clinical practice

Although the evaluation of epigenomic changes in circulating DNA is a promising approach, there are several remaining challenges. Like other newly developed cancer diagnostic assays, validation in larger datasets and standardization are mandatory to ultimately apply this to clinical practice. For disease monitoring, epigenomic signatures could potentially be personalized (rather than a generalized tRCC signature) to improve sensitivity using patient-specific patterns of methylation or chromatin features, similar to mutation-based assays, which enhance accuracy by tracking patient-specific events. A potential intrinsic limitation of epigenomic profiling is that the tumor’s epigenomic landscape is not static. Cancer cells can undergo epigenomic reprogramming during the disease course or in response to treatments (17). Furthermore, TFE3 expression has been observed in a subset of aggressive ccRCCs (18), which might limit the specificity of TFE3-driven epigenomic signatures. However, this dynamic feature of epigenomics may even be advantageous; for instance, should TFE3-targeted therapies become available, epigenomic profiling could potentially be used to determine which tumors are driven by TFE3 activation.

Clinically relevant diagnostic tools and therapeutic decision-making are two sides of the same coin. The information from the test should ultimately be translatable into guiding effective treatment decisions, such as pursuing adjuvant/neoadjuvant treatment (19) and specific therapy selection (20). Given a potentially larger number of hidden tRCC cases (10), this circulating epigenomic identification approach may ultimately improve the diagnosis of this disease, which may also increase the opportunity to study this disease more specifically in clinical trials. Further, the correct diagnosis of RCC subtypes will help eliminate unwanted (and unaccounted for) heterogeneity in clinical trials, where tRCC is accidentally included in a trial that focuses on a different histology (like ccRCC), potentially impacting the results (21, 22). Therefore, there is tremendous potential to include this epigenomic screening of ctDNA approach in future clinical trials and, if validated in larger scale studies, ultimately in clinical practice.

## Conclusions

In summary, Garinet et al. (15) have provided a promising framework to diagnose tRCC based on epigenomic information from ctDNA. Their approach could potentially be applied to other malignancies with few genetic mutations. For clinical application, prospective clinical trials are needed for validation, but ultimately, incorporating epigenomic profiling into liquid biopsies may identify more patients with this uncommon malignancy and enable better treatment selection and monitoring.

## Version 1 02/02/2026

Electronic publication

## References

[1] Ma L (2024). Liquid biopsy in cancer current: status, challenges and future prospects. Signal Transduct Target Ther.

[2] Powles T ctDNA-guided adjuvant atezolizumab in muscle-invasive bladder cancer. N Engl J Med.

[3] Zill OA (2018). The landscape of actionable genomic alterations in cell-free circulating tumor DNA from 21,807 advanced cancer patients. Clin Cancer Res.

[4] Lasseter K (2020). Plasma cell-free DNA variant analysis compared with methylated DNA analysis in renal cell carcinoma. Genet Med.

[5] Pal SK (2017). Evolution of circulating tumor DNA profile from first-line to subsequent therapy in metastatic renal cell carcinoma. Eur Urol.

[6] Correa AF (2024). Association of circulating tumor DNA with patient prognosis in surgically resected renal cell carcinoma. Oncologist.

[7] Nuzzo PV (2020). Detection of renal cell carcinoma using plasma and urine cell-free DNA methylomes. Nat Med.

[8] Baca SC (2023). Liquid biopsy epigenomic profiling for cancer subtyping. Nat Med.

[9] Sun G (2021). Integrated exome and RNA sequencing of TFE3-translocation renal cell carcinoma. Nat Commun.

[10] Bakouny Z (2022). Integrative clinical and molecular characterization of translocation renal cell carcinoma. Cell Rep.

[11] Sukov WR (2012). TFE3 rearrangements in adult renal cell carcinoma: clinical and pathologic features with outcome in a large series of consecutively treated patients. Am J Surg Pathol.

[12] Marcon J (2020). Comprehensive genomic analysis of translocation renal cell carcinoma reveals copy-number variations as drivers of disease progression. Clin Cancer Res.

[13] Chen X (2015). Newly designed break-apart and ASPL-TFE3 dual-fusion FISH assay are useful in diagnosing Xp11.2 translocation renal cell carcinoma and ASPL-TFE3 renal cell carcinoma: a STARD-compliant article. Medicine (Baltimore).

[14] El Zarif T (2024). Epigenomic signatures of sarcomatoid differentiation to guide the treatment of renal cell carcinoma. Cell Rep.

[15] Garinet S (2026). Cell-free DNA epigenomic profiling enables noninvasive detection and monitoring of translocation renal cell carcinoma. J Clin Invest.

[16] Weber M (2005). Chromosome-wide and promoter-specific analyses identify sites of differential DNA methylation in normal and transformed human cells. Nat Genet.

[17] Feinberg AP, Levchenko A (2023). Epigenetics as a mediator of plasticity in cancer. Science.

[18] Lee HJ (2018). TFE3 translocation and protein expression in renal cell carcinoma are correlated with poor prognosis. Histopathology.

[19] Rini BI (2025). Circulating kidney injury molecule-1 (KIM-1) and association with outcome to adjuvant immunotherapy in renal cell carcinoma. Ann Oncol.

[20] Tao J (2023). Liquid biopsy-informed precision oncology study to evaluate utility of plasma genomic profiling for therapy selection. J Clin Oncol.

[21] Alhalabi O (2023). Immune checkpoint therapy combinations in adult advanced MiT family translocation renal cell carcinomas. Oncologist.

[22] Rahimov F (2025). Common Diseases in Clinical Cohorts—Not Always What They Seem. N Engl J Med.

